# Peripartum Takotsubo Cardiomyopathy: A Review and Insights from a National Registry

**DOI:** 10.3390/jcdd11020037

**Published:** 2024-01-25

**Authors:** Ravi Vazirani, Emilia Blanco-Ponce, Manuel Almendro Delia, Agustín C. Martín-Garcia, Clara Fernández-Cordón, Aitor Uribarri, Oscar Vedia, Alessandro Sionis, Jorge Salamanca, Miguel Corbí-Pascual, Alberto Pérez-Castellanos, Manuel Martínez-Selles, Víctor Manuel-Becerra, Sergio Raposeiras-Roubín, David Aritza-Conty, Javier Lopez-País, Marta Guillén-Marzo, Carmen Lluch-Requerey, Iván J. Núñez-Gil

**Affiliations:** 1 Department of Cardiology, Hospital Clínico San Carlos, 28040 Madrid, Spain; ravi_94@hotmail.es (R.V.); oscarvediacruz@gmail.com (O.V.); 2 Department of Cardiology, Hospital Universitario Arnau de Vilanova, Institut de Recerca Biomedica (IRB), 25198 Lleida, Spain; emiliablancoponce@gmail.com; 3 Department of Cardiology, Hospital Virgen de la Macarena, 41009 Sevilla, Spain; trocor@gmail.com; 4 Department of Cardiology, Complejo Asistencial Universitario de Salamanca, 37007 Salamanca, Spain; agusmg.carlos@gmail.com; 5 Department of Cardiology, Hospital Universitario Gregorio Marañón, 28007 Madrid, Spain; cfernandez.1@alumni.unav.es (C.F.-C.); mmselles@secardiologia.es (M.M.-S.); 6 Department of Cardiology, Hospital de Vall d’Hebron, 08035 Barcelona, Spain; auribarri@gmail.com; 7 Department of Cardiology, Hospital Sant Pau an Creu, 08025 Barcelona, Spain; asionis@santpau.cat; 8 Department of Cardiology, Hospital Universitario de La Princesa, 28006 Madrid, Spain; jsalamancaviloria@gmail.com; 9 Department of Cardiology, Hospital Universitario de Albacete, 02006 Albacete, Spain; miguelcorbi@hotmail.com; 10 Department of Cardiology, Hospital Universitario de Son Espases, 07120 Palma, Spain; apcastellanos@hotmail.com; 11 Department of Cardiology, Hospital Universitario de Málaga, 29010 Malaga, Spain; vmbecerram@gmail.com; 12 Department of Cardiology, Hospital Álvaro Cunqueiro, 36312 Vigo, Spain; raposeiras26@hotmail.com; 13 Department of Cardiology, Hospital Universitario de Navarra, 31008 Pamplona, Spain; dconty91@gmail.com; 14 Department of Cardiology, Hospital Universitario de Orense, 32005 Orense, Spain; javierlopezpais@hotmail.com; 15 Department of Cardiology, Hospital Universitario Joan XXIII, 43005 Tarragona, Spain; mgm_82@outlook.es; 16 Department of Cardiology, Hospital Universitario Juan Ramón Jimenez, 21005 Huelva, Spain; carmenlr94@gmail.com; 17 Faculty of Biomedical and Health Sciences, Universidad Europea de Madrid, 28670 Villaviciosa de Odón, Spain

**Keywords:** takotsubo syndrome, prognosis, registry, women, peripartum, pregnancy, cardiomyopathy

## Abstract

Takotsubo syndrome (TTS) during the peripartum period is a relevant cause of morbidity in this population; its clinical course and prognosis, compared to the general TTS population, is yet to be elucidated. Our aim was to analyze the clinical features and prognosis of peripartum TTS in a nationwide prospective specifically oriented registry database and consider the published literature. Peripartum TTS patients from the prospective nationwide RETAKO registry—as well as peripartum TTS patients from the published literature—were included, and multiple comparisons between groups were performed in order to assess for statistically and clinically relevant prognostic differences between the groups. Patients with peripartum TTS exhibit a higher prevalence of secondary forms, dyspnea, atypical symptoms, and echocardiographic patterns, as well as less ST-segment elevation than the general TTS population. In the literature, patients with peripartum TTS had a higher Killip status on admission. TTS during the peripartum period has a higher prevalence of angina and dyspnea, as well as physical triggers, neither of which are related to a worse prognosis. Killip status on admission was higher in the literature for patients with TTS but with excellent mid- and long-term prognoses after the acute phase, despite mostly being secondary forms.

## 1. Introduction

Takotsubo syndrome (TTS) is characterized by transient left ventricular (LV) dysfunction, often triggered by emotional or physical stress. It was first described in Japan in 1990 and [1] it is known to mainly affect postmenopausal women. 

Nevertheless, TTS after cesarean delivery was first reported in 2008 by Muller et al. [2] and, thereafter, some peripartum cases have been reported. Labor pains and surgical stress, which accompany delivery, may contribute to the development of TTS during the peripartum period [3]. In the postpartum period, estrogen levels may be depleted abruptly because of placenta expulsion. In addition, delivery is linked to intense emotional and physical stress, which may lead to a catecholamine surge. The reduced estrogen levels might make the myocardium more susceptible to the catecholamine surge [1].

TTS differential diagnosis in pregnancy includes pulmonary embolism, spontaneous coronary artery dissection (SCAD), coronary artery spasm, ischemic obstructive coronary artery disease, and peripartum cardiomyopathy (PPCM). To date, large retrospective databases [1,3] have focused on comparing peripartum TTS with PPCM but there are no prospective TTS registries assessing the differences between TTS during pregnancy and the puerperium period compared to the standard presentation of this disease. 

Thus, our aim was to analyze the clinical features and prognosis of peripartum TTS in a nationwide prospective specifically oriented registry database, considering the published literature to date.

## 2. Material and Methods

This study complied with the Declaration of Helsinki and was approved by the Institutional Ethics Committee of the coordinator center. All patients provided informed consent.

### 2.1. Review Methods

**Figure 1:**
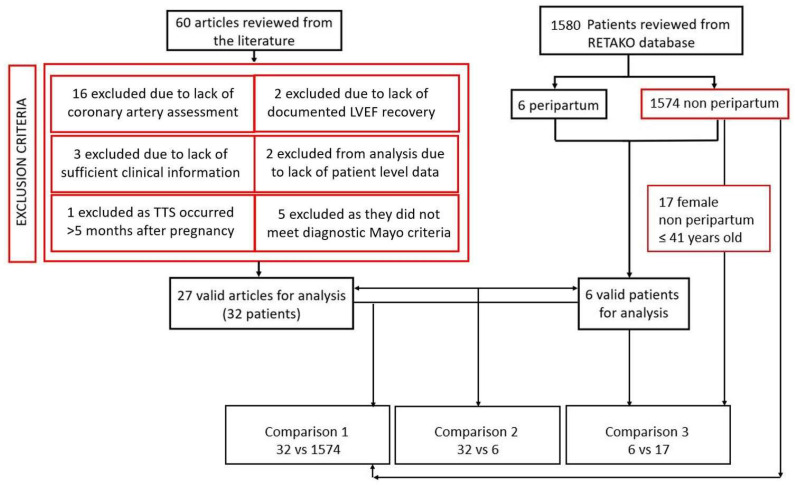
Flowchart summarizing both the literature and the RETAKO dataset review process and selection criteria. A total of 38 peripartum patient-level data cases (32 from the literature and 6 from RETAKO registry) were available for analysis at the end of the review process.

For our study, inclusion criteria were set prior to the literature search and only publications in English were included [4]. Peripartum TTS was defined as such when a patient had transient regional wall motion abnormalities (RWMAs) that extended beyond a single epicardial vascular distribution (e.g., apical, basal, or midventricular ballooning) during pregnancy or within 5 months after delivery, with either electrocardiographic abnormalities or modest cardiac troponin elevation, without coronary artery disease (excluded either with coronary angiography or cardiac CT scan) according to Mayo clinic criteria [5]. Exclusion criteria for peripartum TTS include: proven acute coronary syndrome, SCAD, significant valvular heart disease, previous valve surgery, rheumatic heart disease, other known cardiomyopathy syndromes, and pheochromocytoma or myocarditis [3]. Figure 1 describes the flowchart summarizing the review methodology. A detailed description of the review methods can be found in the Supplementary Material.

### 2.2. Registry Data and Study Definitions

The comparisons were based on data from an ongoing national multicenter TTS registry (RETAKO), supported by the Ischemic Heart Disease and Acute Cardiovascular Care Section of the Spanish Society of Cardiology, starting 1 January 2012. Data were collected at each hospital, anonymized, electronically encrypted, and transferred online to a central database. Its rationale and design have been previously described [6]. Main inclusion criteria required a definitive TTS diagnosis based on the modified Mayo Clinic criteria [5]. Follow-up after hospital discharge was conducted via regular outpatient visits or telephone interviews with patients or relatives. Follow-up outcomes were pre-specified and defined as the first nonfatal TTS recurrence and/or the occurrence of all-cause death. If a pre-specified outcome was observed, review of electronic medical records and consensus of 2 experienced local investigators were mandatory for event adjudication. TTS patients were classified as primary or secondary according to the trigger, with the first consisting of psychological or unidentifiable triggers, and the latter being either physical or mixed [7]. Killip status on admission was also measured, which consists of a classification system extensively validated in patients’ myocardial infarctions that divides the patients into 4 groups according to their clinical profile; it helps to predict mortality and guide treatment decisions, improving patient outcomes [8]. 

The following specific comparison levels between groups were carried out (further details in the results area): (1) The literature peripartum TTS vs. RETAKO general cohort; (2) RETAKO peripartum TTS vs. the literature peripartum TTS patients; and (3) RETAKO peripartum TTS vs. young non-peripartum RETAKO female patients (up to 41 years old). The latter was performed in order to assess for differences between young pregnant and non-pregnant females with TTS within the RETAKO cohort. 

### 2.3. Statistical Analysis

Clinical data, echocardiographic findings, and clinical outcomes are presented with standard descriptive statistics—mean ± standard deviation (SD) or median ± interquartile range (IQR)—when needed. Comparisons between groups were performed using Pearson’s chi-squared for qualitative variables and Student’s *t*-test or ANOVA tests when appropriate, for continuous variables. A 2-tailed *p* < 0.05 was considered as statistically significant. Statistical analyses and graphing were performed using Office package 365 (Microsoft, Redmon, Washington, DC, USA) and SPSS software v25 (IBM, Chicago, IL, USA).

## 3. Results

Baseline clinical data from both RETAKO peripartum TTS and the literature peripartum TTS are described in Supplementary Table S1. None of the included patients from the literature peripartum TTS group had cardiovascular complications during their described follow-up period; in addition, there were no recurrences and/or complications during the described follow-up for any patient included in this analysis.

When compared to the RETAKO general cohort, women with peripartum-related TTS were younger (30.6 vs. 68.1 years; *p* < 0.001) and less commonly hypertensive (*p* < 0.001) and hyperlipidemic (*p* < 0.001) (Table 1). 

The left ventricular ejection fraction (LVEF) was lower in the literature peripartum TTS group (34.9% vs. 42.4%, *p* = 0.001) with less prevalence of apical ballooning (37.5%, *p* < 0.001) on the echocardiogram and higher incidence of atypical patterns (62.5%, *p* = 0.001) when compared to the RETAKO general cohort.

Cardiogenic shock (Killip IV status) during hospital stay was more common in the literature peripartum TTS group (*p* < 0.001) with a greater need for vasoactive support, intra-aortic balloon pump (IABP) support, and mechanical ventilatory support. There were neither higher cumulative incidences of cardiovascular complications (defined as death, readmissions in cardiology, and/or TTS recurrences) (24.2% vs. 0%, *p* = 0.001), nor in all-cause mortality (13.3% vs. 0%, *p* = 0.03) in the literature peripartum TTS group during their follow-up; with a median of 165 days (IQR 33–353) for the peripartum TTS and 581 days (IQR 155–1588) for the RETAKO general cohort.

Comparisons between the RETAKO peripartum TTS and the literature peripartum TTS populations are depicted in Table 2. 

Clinical characteristics were similar between both groups, with palpitations (*p* = 0.026) and renal failure (*p* = 0.034) being slightly more common in the RETAKO peripartum TTS group. Nevertheless, cardiogenic shock was significantly higher in the literature peripartum TTS group (53.8% vs. 16.7%, *p* = 0.016). No differences were found in the TTS presentation, as secondary forms were more common than primary in both groups (83.3 vs. 80.8%, *p* = 0.885); neither in ECG abnormalities on admission, nor in follow-up complications or deaths (0% in both groups for both variables), with a greater follow-up in the RETAKO peripartum TTS group compared to the published literature: 1353.8 days (IQR 296.2–2062.7) vs. 131.8 days (IQR 27.7 vs. 180.0).

Comparisons between the RETAKO peripartum TTS group and young non-peripartum RETAKO female patients (both with the older patient being 41 years old) from the RETAKO population are depicted in Table 3. 

Clinical characteristics were similar in both groups, without statistically significant differences in the trigger; even though primary forms were more numerically more common in the young non-peripartum RETAKO female patients (16.7% vs. 56.3%, *p* = 0.097) with a trend towards atypical symptoms, such as angina (16.7% vs. 62.5%, *p* = 0.056). Killip status on admission showed no overall differences (*p* = 0.56), neither did LVEF (38.83% vs. 40.27%, *p* = 0.857), nor the TTS pattern (*p* = 0.40). There was a non-significant trend of less ST-segment elevation in the RETAKO peripartum TTS group (0% vs. 43.7%, *p* = 0.124), without differences in deaths and follow-up complications in both groups (*p* = 0.54 in the former) with a similar median follow-up for the peripartum (1353 days (IQR 296.2–2062.7)) and the non-peripartum groups (1297 days (IQR 332.2–2399.3) (*p* = 0.94)).

## 4. Discussion

The present study assesses, to the best of our knowledge, the largest cohort published with TTS in a peripartum setting to date. We provide a pooled comparison with peripartum TTS from the literature and the RETAKO registry with the overall population of patients with TTS and, moreover, with a similar sample of non-peripartum young TTS females. 

Our main findings include: patients with peripartum TTS exhibit a higher prevalence of dyspnea with atypical symptoms and echocardiographic patterns, as well as less ST-segment elevation than the “standard” TTS profile. The literature peripartum TTS patients had a higher Killip status on admission but with excellent mid- and long-term prognosis after the acute phase.

The discussion will focus on the disparities between the multiple comparisons performed along the three groups, as the similarities described in Table 1, Table 2 and Table 3 such as greater age and classical cardiovascular risk factors (CVRFs) in the RETAKO general cohort—as well as deaths and follow-up complications—are consistent with the previous literature [9] and have been thoroughly analyzed and described in a previous article [6].

Symptoms were more commonly atypical in the peripartum population compared with the general RETAKO cohort, with less angina and more dyspnea on admission in the former. This finding is also consistent, especially for the absence of angina, with the comparison between RETAKO peripartum TTS and young non-peripartum RETAKO female patients, not being statistically significant for the absence of angina (*p* = 0.056), possibly because the test was underpowered due to the low number of patients included in both groups (16 and 6, respectively). Probably, this fact is derived from a lower LVEF. Interestingly, dyspnea at admission has been associated [9] with a higher degree of comorbidity burden and worse long-term prognosis in a large cohort of the GEIST registry with a mean age of 72 years and 20% of pulmonary disease. Our findings suggest that dyspnea is neither a marker of greater comorbidity nor a marker of worse long-term prognosis in the peripartum population; we hypothesize that the dyspnea mechanism (acute pulmonary congestion, not underlying pulmonary disease) and the younger age are the great outcome contributors [6]. The lower cardiovascular risk profile in the general RETAKO peripartum TTS might reflect peripartum TTS patients being younger.

The TTS trigger exhibited clear differences between the RETAKO general cohort, in which primary forms are more common (psychological or non-identifiable); and the peripartum group, in which secondary forms (physical or mixed) predominated. The latter was mainly in the context of a cesarean section (CS), peripartum physical stress, or comorbidities in this setting, as highlighted in Supplementary Table S1. These findings are not in line with the published literature for regular TTS [10], which associated worse short- and long-term prognoses to physical triggers in general and the ones in which hypoxia is the trigger in particular. In the aforementioned study, a physical trigger, an age >70 years old, and diabetes mellitus were significant predictors of long-term mortality. Thus, only one of these predictors (the physical trigger) is predominant in our peripartum population, with no deaths or long-term complications. This finding could be related to the fact that the “peripartum setting” is a transient situation and suggests that conclusions drawn from studies with older patients might not apply to younger ones and that death is likely mainly driven by age and comorbidities. 

The pathophysiological causes of TTS in this particular population are yet to be elucidated; Citro et al. [11] hypothesize that the sum of endogenous (intense emotional and physical stress related to labor and CS) and exogenous (anesthesia, uterotonics) catecholaminergic stimulation, along with the abrupt drop in estrogen-induced protective cardiovascular effects after placental delivery, might act as underlying pathophysiologic mechanisms. 

Atypical TTS echocardiographic patterns (midventricular and basal) were more common in younger patients (without statistical significance between peripartum and non-peripartum) compared to the RETAKO general cohort; hence representing that atypical forms are more common in younger patients, irrespective of their peripartum status, which is consistent with the published literature [12]. Also, the higher prevalence of atypical patterns would explain the absence of left ventricular outflow tract (LVOT) obstruction described in previous studies [13].

Electrocardiographic alterations also exhibited statistically significant and clinically relevant findings: peripartum TTS showed less frequent ST-segment elevation on admission EKG compared to the general RETAKO cohort and the young non-peripartum RETAKO female patients. Consistently, we found no differences between both peripartum studied populations. From the available data, ST-segment elevation is less common in peripartum TTS, with initial T-wave inversion being the most common initial repolarization abnormality between all the compared groups. Better long-term prognosis and an absence of deaths in the peripartum group might be associated with the absence of ST-segment elevation on admission, described as a protective factor in a previous work [14]. All patients were in sinus rhythm in the first admission ECG, except for two cases, in which the initial rhythm was ventricular tachycardia [15] and ventricular fibrillation [16].

Follow-up complications (readmissions in cardiology and TTS recurrences) and deaths were more common in the general RETAKO cohort compared to the peripartum group. When assessing differences between both peripartum populations, no events were found, neither in the RETAKO peripartum TTS group nor the literature peripartum TTS, with both groups agreeing on a potentially favorable prognosis. However, one could argue that events in the published literature might be affected by the so-called “immortal time bias”, as the mean follow-up in days (131.8 vs. 1353.8, *p* = 0.003) was significantly shorter in this group and events could appear during the follow-up; however, the fact that no events were registered in the RETAKO peripartum TTS population and young non-peripartum RETAKO female population, with a median follow-up of 1353 and 1297 days, respectively (*p* = 0.94), favors the reasoning that recurrences and readmission are more common in older female patients with primary forms, which is consistent with the published literature [17]. 

Killip status on admission showed consistent differences in the literature peripartum TTS group compared to the rest of the groups: the general RETAKO cohort, in which the differences are mainly driven by a Killip IV status (48.8% vs. 9.8%, *p* < 0.001) and are supported by statistically significant differences in vasoactive support, IABP, mechanical ventilatory support, and lesser LVEF on admission. This finding remains constant when comparing it to the RETAKO peripartum TTS population, with a Killip IV status of 53.8% vs. 16.7%, respectively (*p* = 0.016). The excellent medium- and long-term prognoses despite the high initial Killip status seem to diverge from the recent evidence in MINOCA patients [18]; where a higher Killip status on admission was an independent predictor of MACE occurrence during follow-up. Even though our findings could suggest that TTS has a more aggressive clinical course or that peripartum patients might be over-treated with pharmacological, mechanical circulatory, and ventilatory support, the comparison of Killip IV status between the peripartum and young non-peripartum RETAKO female patients yields statistically non-significant findings (16.7% vs. 25%, *p* = 0.56). We interpreted the huge disparities between the RETAKO peripartum TTS population and the literature peripartum TTS population as a possible publication bias, as both peripartum populations are similar on all traits but cardiogenic shock (Killip IV) on admission, and sicker peripartum patients requiring extracorporeal membrane oxygenation (ECMO) [19], left ventricular assist device (LVAD) [20], IABP [21], and orotracheal intubation [22] might have aroused interest for clinical publication.

Furthermore, new evidence [23] regarding IABP use in TTS with cardiogenic shock (CS) in the GEIST registry depicts a totally different clinical profile of the TTS patient that suffers from CS and requires IABP, which includes being male with diabetes mellitus and with biventricular involvement. This clinical profile from three multicenter and prospective registries is consistent with the classical CS profile in TTS [8] and differs from the one obtained with the published literature peripartum group, thus favoring publication bias as a plausible explanation for the higher incidence of CS in the published literature.

This finding highlights the importance of prospective, observational, national [6], and international [9] multicentric registries with independently audited databases to provide unbiased data for adequate clinical comparison and meaningful interpretation of the data. Provided the limitations of the low number of patients used for comparison, peripartum TTS patients in the RETAKO registry do not have a worse Killip status on admission than both young non-peripartum RETAKO females and the general RETAKO cohort. 

The published literature on TTS in the peripartum period is scarce, only two large retrospective analyses have been performed comparing it with peripartum cardiomyopathy: Kim et al.’s [3] population is similar to ours in baseline characteristics, with a higher degree of complications in the peripartum TTS group—40% bleeding complications and 50% orotracheal intubation, without deaths in a 3-year follow-up period. Yang et al.’s [1] population is, despite describing a different racial group, comparable to our data. Both showed excellent long-term outcomes but are subjected to bias inherent in retrospective cohorts and the risk of misdiagnosing peripartum cardiomyopathy by TTS in some cases in the latter study. 

## 5. Limitations

First, we need to take into account the restrictions of an observational design. Regarding the registry, it is possible that some incident cases in the participating hospitals have not been diagnosed, have been admitted to other specialties, have not undergone catheterization, have not agreed to participate, or have not been reported to the Registry. Therefore, the potential risk of bias warrants that the findings of the present study must be considered with caution. A low number of patients was used for comparison between the peripartum groups.

Follow-up was shorter in the peripartum population retrieved from the literature, which might underrepresent relevant clinical events occurring later in time. In this group, a publication bias could not be discarded either. Surprisingly, despite the frequent use of IABP or ventricular assist devices, no deaths were recorded in this group, highlighting the possibility of an underreporting of patients with negative outcomes in the literature. 

The management of all these patients was the current management at that time in every hospital and depended on the local physician. So, in this way, we believe our results reflect closely real clinical practice. 

Currently, there is not a gold standard to differentiate between PPCM and TTS in the peripartum period, Mayo clinic criteria [5] were strictly applied, and the results were similar to two of the largest peripartum TTS cohorts [1,3].

## 6. Conclusions

TTS during the peripartum period shows a higher prevalence of atypical symptoms on admission. Atypical echocardiographic TTS patterns are more common in the peripartum group, which exhibits less ST-segment elevation than the “standard” TTS group as well. Dyspnea on admission and physical triggers are more common in peripartum TTS but do not entail a worse mid-term prognosis in this population. Admission Killip status is higher with a lower LVEF in the peripartum population but with excellent mid- and long-term prognoses after the acute phase.

## Figures and Tables

**Table 1:** A comparison between TTS in the general cohort and during the literature peripartum TTS (of which 32 patients had analyzable data for this table).

		Takotsubo Syndrome (RETAKO General Cohort) (n = 1574)	The Literature Peripartum Takotsubo Syndrome (n = 32)	*p* Value
Age (years)		68.1 ± 30.6	35 ± 5	<0.001
Female		1355 (86.1%)	31 (100%)	0.172
Hypertension		1015 (67.4%)	4 (12.9%)	<0.001
Dislipidaemia		794 (50.5%)	2 (6.5%)	<0.001
Diabetes Mellitus		394 (25%)	0 (0%)	0.068
Smoker/Ex-smoker		408 (27.2%)	2 (6.4%)	0.182
Obesity		214 (14.7%)	3 (9.7%)	0.433
TTS Trigger	Secondary form	543 (34.5%)	26 (81.3%)	<0.001
Symptoms	Angina *	938 (64.3%)	9 (28.1%)	<0.001
	Dyspnea	762 (41.3%)	23 (76.7%)	<0.001
	Palpitations	112 (7.6%)	3 (9.7%)	0.065
	Syncope	124 (8.4%)	0 (0%)	0.093
In-hospital complications	Stroke	41 (2.8%)	2 (6.5%)	0.228
	Bleeding	49 (3.3%)	1 (3.2%)	0.974
	Renal failure	170 (11.6%)	1 (3.2%)	0.146
Maximum Killip status	I	940 (59.7%)	12 (38.7%)	<0.001
	II	238 (15.1%)	1 (3.2%)	
	III	136 (8.6%)	3 (9.7%)	
	IV	155 (9.8%)	15 (48.4%)	
Vasoactive support		189 (30.8%)	15 (53.6%)	0.019
IABP		21 (4.1%)	4 (14.3%)	0.008
MVS		108 (19.5%)	10 (37%)	0.027
LVEF		42.14 ± 11.80	34.93 ± 10.86	0.001
Takotsubo pattern	Typical	1173 (74.6%)	12 (37.5%)	<0.001
	Atypical	400 (25.4%)	20 (62.5%)	0.001
ECG abnormalities	ST-segment elevation	746 (51.9%)	7 (22.6%)	0.001
	ST-segment depression	198 (13.9%)	3 (9.7%)	0.503
	Initial T-wave inversion	569 (39.8%)	11 (35.5%)	0.623
Follow-up complications		313 (24.2%)	0 (0%)	0.001
Deaths		209 (13.3%)	0 (0%)	0.030
Follow-up		165, 33–353	581, 155–1588	0.001

Follow-up time is expressed as days (median) and interquartile range (IQR). Patients from RETAKO included in the peripartum group were excluded from RETAKO general population for this analysis. Follow-up complications were considered a composite between readmissions in cardiology and/or TTS recurrences. Regarding the TTS pattern, typical forms included apical involvement only, the rest were considered atypical. * Calculated over 1459 with available data on the RETAKO registry. Abbreviations: CP = chest pain; IABP = intra-aortic balloon pump; LVEF = left ventricular ejection fraction; MVS = mechanical ventilatory support. Percentages are given over available patients in each group for a certain variable. The form of TTS was more commonly secondary (*p* < 0.001) with atypical symptoms such as dyspnea (*p* < 0.001). The literature peripartum TTS group had a lower prevalence of ST-segment elevation on the admission ECG (51.9% vs. 22.6%, *p* < 0.001), whereas T-wave inversion was equally found in both groups on admission ECG (39.8% vs. 35.5%, *p* = 0.623).

**Table 2:** Differences between the RETAKO peripartum TTS and the literature peripartum TTS populations. The comparison included 6 patients from the RETAKO database and 32 from the literature review.

		RETAKO Peripartum Takotsubo Syndrome (n = 6)	The Literature Peripartum Takotsubo Syndrome (n = 32)	*p* Value
Age (years)		38.3 ± 2.3	34.2 ± 5.0	0.056
Hypertension		1 (16.7%)	4 (15.4%)	0.938
Dislipidaemia		1 (16.7%)	1 (3.8%)	0.242
Diabetes Mellitus		0 (0%)	0 (0%)	n/a
Smoker/Ex-smoker		1 (16.7%)	1 (3.8%)	0.098
Obesity		0 (0%)	4 (15.4%)	0.304
TTS Trigger	Secondary form	5 (83.3%)	21 (80.8%)	0.885
Symptoms	Angina	1 (16.7%)	8 (30.8%)	0.489
	Dyspnea	5 (100%)	19 (73.1%)	0.197
	Palpitations	2 (33.3%)	1 (3.8%)	0.026
	Syncope	0 (0%)	0 (0%)	
In-hospital complications	Stroke	1 (16.7%)	1 (3.8%)	0.242
	Bleeding	0 (0%)	1 (3.8%)	0.625
	Renal failure	1 (16.7%)	0 (0%)	0.034
	Embolic events	0 (0%)	0 (0%)	
Maximum Killip status	I	2 (33.3%)	11 (42.3%)	0.016
	II	1 (16.7%)	0 (0%)	
	III	2 (33.3%)	1 (3.8%)	
	IV	1 (16.7%)	14 (53.8%)	
LVEF		38.8 ± 13.6	35 ± 11.2	0.475
TTS pattern	Typical	2 (33.3%)	10 (38.5%)	0.815
ECG abnormalities	ST-segment elevation	0 (0%)	7 (26.9%)	0.15
	ST-segment depression	1 (16.7%)	2 (7.7%)	0.497
	Initial T-wave inversion	3 (50%)	8 (30.8%)	0.371
Follow-up complications		0 (0%)	0 (0%)	
Deaths		0 (0%)	0 (0%)	
Follow-up		1353.8 (296.2–2062.7)	131.8 (27.7–180.0)	0.003

Follow-up time is expressed as days (median) and interquartile range (IQR).

**Table 3:** Differences between RETAKO peripartum TTS group and young non-peripartum RETAKO female patients. The comparison included 6 pregnant and 16 non-pregnant patients from the RETAKO database.

		RETAKO Peripartum Takotsubo Syndrome (n = 6)	Young Non-Peripartum RETAKO Female Patients (n = 17)	*p* Value
Age (years)		38.3 ± 2.3	38.2 ± 4.0	0.934
Hypertension		1 (16.7%)	2 (13.3%)	0.844
Dislipidaemia		1 (16.7%)	2 (12.6%)	0.636
Diabetes Mellitus		0 (0%)	1 (6.3%)	0.531
Smoker/Ex-smoker		1 (16.7%)	7 (43.7%)	0.623
Obesity		0 (0%)	0 (0%)	
TTS Trigger	Secondary form	5 (83.3%)	7 (41.2%)	0.076
Symptoms	Angina	1 (16.7%)	10 (62.5%)	0.056
	Dyspnea	6 (100%)	7 (63.6%)	0.35
	Palpitations	2 (33.3%)	1 (6.7%)	0.375
	Syncope	0 (0%)	2 (13.3%)	0.906
In-hospital complications	Stroke	1 (16.7%)	0 (0%)	0.627
	Bleeding	0 (0%)	0 (0%)	
	Renal failure	1 (16.7%)	0 (0%)	0.10
	Embolic events	0 (0%)	0 (0%)	
Maximum Killip status	I	2 (33.3%)	7 (43.7%)	0.56
	II	1 (16.7%)	3 (18.8%)	
	III	2 (33.3%)	1 (6.3%)	
	IV	1 (16.7%)	4 (25%)	
LVEF		38.83 ± 13.64	40.27 ± 17.10	0.857
TTS pattern	Typical	2 (33.3)	9 (52.9%)	0.40
ECG abnormalities	ST-segment elevation	0 (0%)	7 (43.7%)	0.124
	ST-segment depression	1 (16.7%)	5 (33.3%)	0.819
	Initial T-wave inversion	3 (50%)	5 (33.3%)	0.831
Follow-up complications		0 (0%)	0 (0%)	
Deaths		0 (0%)	1 (6.3%)	0.54
Follow-up		1353,296.2–2062.7	1297,332.2–2399.2	0.94

Follow-up time is expressed as days (median) and interquartile range (IQR).

## Data Availability

The data is available upon reasonable request to the corresponding author.

## Supplementary Materials

The following supporting information can be downloaded at: https://www.mdpi.com/article/10.3390/jcdd11020037/s1, Review Methods S1: Review methods and Supplemental references; Table S1: Baseline characteristics and clinical course from the analyzed peripartum population. References [1,3] are cited in the Supplementary Materials.
