# Supplementary material for: Peripartum Takotsubo Cardiomyopathy: A Review and Insights from a National Registry
Source: J Cardiovasc Dev Dis. 2024 Jan 25;11(2):37. doi: 10.3390/jcdd11020037 (PMC10889154; doi:10.3390/jcdd11020037)
Supplement: Supplementary file 1 [file jcdd-11-00037-s001.zip › jcdd-2826376-supplementary.pdf]

Table 1.

Patient nº	Source	Age	Race	CVRFs	Hypertensive states in pregnancy	Trigger (physical/stress/not clarified)	Initial LVEF	Recovery of LVEF**	Pattern	Initial symptoms	Repolarization abnormalities on admission ECG***	Vasoactive support during/after delivery	Complications during acute phase	Follow-up (days)
1	RETAKO	41	White	Yes	Hypertension in pregnancy	Psychological (sadness)	45	>3	Apical	Angina	TWI, STE	No	No	5383
2	RETAKO	40	White	No	No	Physical (puerperal fever)	38	>3	Global	Dyspnea	No	No	No	787
3	RETAKO	36	Black	No	No	Physical (Autoimmune nephropathy and abortion)	44	1	Apical	Angina and dyspnea	STD	Yes	Acute pulmonary edema, electromechanical dissociation, OTI	236
4	RETAKO	35	South-American	No	No	Physical (caesarean-section)	55	3	Basal	Dyspnea	TWI	No	No	956
5	RETAKO	39	White	No	No	Physical (caesarean-section)	48	>3	Midventricular	Dyspnea	TWI	No	Acute pulmonary edema after caesarean section	317
6	RETAKO	41	White	No	No	Physical (ischaemic stroke in puerperium)	40	1	Basal	No	No	No	No	323
7	Tomida et al.	35	Asian (japanese)	No	No	Physical (caesarean-section)	20	3	Midventricular	Dyspnea	No	Yes	Cardiogenic shock	150
8	Ghariani et al	30	Arab	No	No	Physical (uterine bleeding)	25	1	Basal	Dyspnea	No	Yes	OTI	21
9	Gabarre et al.	38	White	No	No	Physical (HELLP syndrome)	45	1	Basal	Headache, atypical pain	No	No	HELLP	90
10	Kandah et al	35	Black	Yes	Pre-eclampsia	Physical (postpartum stress)	60	1	Apical	Angina and dyspnea	No	No	No	N/A
11	Jih et al	33	White	No	No	Physical (post-abortion sepsis)	32	N/A	Global	Dyspnea	VT	Yes	No	42
12	Kourkoveli et al	33	White	No	No	Physical (caesarean-section)	35	1	Basal	Dyspnea and atypical chest pain	No	No	Acute pulmonary edema after caesarean section	30
13	Horie et al.	44	Asian (japanese)	Yes	Pre-eclampsia	Not clarified	31	1	Apical	Dyspnea and anasarca	TWI	Yes	Acute pulmonary edema	180
14	Oindi et al.	35	Asian (indian)	No	Pre-eclampsia	Physical (pre-eclampsia)	48	1	Apical	Angina and dyspnea	STE	No	No	180
15	Ruiz et al.	39	White	No	No	Physical (caesarean-section)	48	3	Basal	Dyspnea	TWI	No	No	90
16	Ledakowicz-Polak et al.	36	White	No	No	Physical (caesarean-section)	30	1	Basal	Dyspnea	STE, STD	Yes	Acute pulmonary edema after caesarean section	365
17	Hamandachi et al	30	White	No	No	Not clarified	27	1	Basal	Atypical chest pain	TWI	No	No	30
18	Wu et al	33	Asian (japanese)	No	No	Physical (postpartum haemorrhage)	30	1	Apical	Cardiogenic shock	STE	Yes	Yes, ECMO and LVAD due to cardiogenic shock	365

Table 1. *Continuation*

Patient nº	Source	Age	Race	CVRFs	Hypertensive states in pregnancy	Trigger (physical/stress/not clarified)	Initial LVEF	Recovery of LVEF**	Pattern	Initial symptoms	Repolarization abnormalities on admission ECG***	Vasoactive support during/after delivery	Complications during acute phase	Follow-up (days)
19	Minatoguchi et al	24	Asian (japanese)	No	No	Physical (caesarean-section)	46	1	Basal	Dyspnea	No	No	No	N/A
20	Parodi et al	32	White	No	No	Physical (anaesthesia during caesarean section)	30	1	Apical	Dyspnea	TWI	Yes	Pulmonary edema and IABP	365
21	Yaqub et al	32	White	No	No	Psychological (postpartum depression)	45	3	Apical	Angina	STE	No	No	42
22	Citro et al	42	White	No	No	Physical (ergonovine injection)	30	1	Midventricular	Angina	TWI	Yes	Respiratory distress and OTI	365
23	Sahng Lee et al	30	Asian (japanese)	No	No	Physical (caesarean section)	35	1	Basal	Dyspnea and atypical chest pain	TWI	No	No	14
24	Sato et al	41	Asian (japanese)	No	No	Physical (caesarean section)	28	1	Apical	Dyspnea	STE	Yes	Acute pulmonary edema and OTI	N/A
25	Jo et al	37	Asian (japanese)	No	No	Physical (caesarean section)	14	3	Apical	Angina and dyspnea	TWI	Yes	ECMO	180
26	D´Agostino et al	42	White	Yes	No	Psychological (postraumatic stress)	42	1	Apical	Angina and dyspnea	STE	No	No	60
27	Citro et al	35	White	No	No	Physical (vaginal delivery)	30	1	Midventricular	Dyspnea	TWI	Yes	IABP due to hypotension	180
28	Citro et al	24	White	No	No	Physical (caesarean section)	30	1	Midventricular and basal	Dyspnea and palpitations	STD	Yes	IABP and OTI	180
29	Ashrafpoor et al	34	White	No	No	Physical (caesarean section)	36	3	Midventricular and basal	Dyspnea and atypical chest pain	No	No	No	90
30	D´Amato et al	32	White	No	No	Psychological (anxiety)	22	1	Apical	Cardiac arrest	VF	Yes	Abortion due to TTS	N/A
31	Muller et al	30	White	No	No	Physical (postpartum stress)	N/A	1	Basal	Angina	STD	No	No	300
32	Zdanowicz et al	32	White	Yes	Hypertension in pregnancy	Physical (spinal anaesthesia)	56	1	Midventricular	Angina	STE	Yes	IABP and stroke	90
33	Kim et al (n=10)	31,3*	Asian (korean)	N/A	Eclampsia (n=4)	All 10 had physical trigger	31.8	70% in 1st month	5 patients with atypical forms	N/A	-	-	ECMO, major bleeding and OTI	1095
34	Yang et al (n=16)	35*	Asian (korean)	N/A	Hypertensive pregnancy state (n=9)	-	37	100% within the 1st month	11 patients with atypical forms	N/A	-	-	-	-

Supplementary Table S1. Baseline characteristics and clinical course from the analyzed peripartum population (the literature peripartum TTS and RETAKO peripartum TTS). * Mean of combined data from the studies of Kim et al (3) and Yang et al (1). **LVEF recovered within the first 1 month is labeled as "1"; within the first 3 months as "3"; and from the third to sixth month as ">3". Abbreviations: CVRFs = cardiovascular risk factors; DIC = disseminated intravascular coagulopathy; ECMO = extracorporeal membrane oxygenation; HELP = hemolysis, elevated liver enzymes, and low platelet count; IABP = intra-aortic balloon pump; LVAD = left ventricular assist device; LVEF = left ventricular ejection fraction; N/A = non-applicable; OTI = orotracheal intubation; TWI = T-wave inversion; STD = ST-segment depression; STE = ST-segment elevation; VT = ventricular tachycardia; VF = ventricular fibrillation.
